# Nine‐Year Recurrence‐Free Survival in Gastric Signet‐Ring Cell Carcinoma: A Case Report of Laparoscopic Radical Resection Combining Long‐Term Systematic Therapy and Metformin Enteric‐Coated Tablets

**DOI:** 10.1155/carm/6766219

**Published:** 2026-05-13

**Authors:** Lingzhi Liao, Guogui Wang, Yifan Li, Dan Zou, Jing Liu, Bin Ma, Jingwei Ma, Yali Li, Yile Qi, Jiahui Ma, Hao Chen

**Affiliations:** ^1^ The Second Hospital & Clinical Medical School, Lanzhou University, Lanzhou, China, lzu.edu.cn; ^2^ The First Hospital & Clinical Medical School, Lanzhou University, Lanzhou, China, lzu.edu.cn; ^3^ Gansu Provincial Key Laboratory of Environmental Oncology, The Second Hospital & Clinical Medical School, Lanzhou University, Lanzhou, China, lzu.edu.cn; ^4^ Department of Surgical Oncology, The Second Hospital & Clinical Medical School, Lanzhou University, Lanzhou, China, lzu.edu.cn; ^5^ The Key Laboratory of Humanized Animal Models, The Second Hospital & Clinical Medical School, Lanzhou University, Lanzhou, China, lzu.edu.cn; ^6^ The Cancer Center of Shanghai General Hospital, Shanghai Jiao Tong University School of Medicine, Shanghai, 200080, China, shsmu.edu.cn

**Keywords:** gastric signet-ring cell carcinoma (GSRCC), metformin, recurrence-free survival (RFS)

## Abstract

Gastric signet‐ring cell carcinoma (GSRCC) represents 10%–14% of all gastric malignancies. The overall prognosis for GSRCC is poorer compared to other types of gastric cancer, as it is characterized by a high degree of malignancy, rapid progression, and early metastasis (particularly peritoneal metastasis). Current therapies combining surgery and chemotherapy are challenged by tumor invasiveness, drug resistance, and limited targeted options. Herein, we present the case of a 60‐year‐old male diagnosed with GSRCC who underwent laparoscopic distal radical gastrectomy with D2 lymphadenectomy. Postoperative histopathological analysis confirmed a mixed tumor composition comprising 60% GSRCC and 40% moderately differentiated adenocarcinoma. Following surgery, the patient was administered adjuvant therapy with oxaliplatin and S‐1 and metformin enteric‐coated tablets for 6 months, followed by 42‐month maintenance therapy regimen combining oral S‐1 and apatinib and metformin enteric‐coated tablets. Over 9 years of follow‐up, the patient demonstrated sustained recurrence‐free survival (RFS) and achieved clinical recovery. This case underscores the potential of individualized adjuvant therapy in addressing the aggressive biological characteristics of GSRCC and offers a valuable reference for strategies to minimize recurrence in GSRCC patients. Trial Registration: ClinicalTrials.gov identifier NCT01101438

## 1. Introduction

Gastric cancer presents significant regional clustering characteristics in East Asia, with China, Japan, and South Korea accounting for more than 60% of the global cases [1, 2]. Gastric signet‐ring cell carcinoma (GSRCC) represents a distinct diffuse‐invasive histopathological entity, comprising approximately 10%–14% of all cases [3]. Compared with non‐signet‐ring cell carcinoma (non‐SRCC), signet‐ring cell carcinoma (SRCC) predominantly affects women and younger patients [4]. The 5‐year overall survival (OS) rate of early GSRCC can reach 72%–89% [5]. A meta‐analysis encompassing 21 studies revealed that GSRCC demonstrates a survival advantage in early‐stage (HR = 0.60, *p* < 0.001), while exhibiting significantly worse outcomes in advanced‐stage (HR = 1.18, *p* < 0.001) [6], attributed to its aggressive invasiveness, early metastasis, and poor chemotherapy response, particularly in advanced stages [7]. The peritoneal metastasis rate of GSRCC patients was significantly higher than that of non‐GSRCC (43% vs. 22%, *p* < 0.001), especially in T3/T4 tumors accounting for 61% [5].

According to current guidelines, the management of GSRCC should follow stage‐specific strategies. For early‐stage disease, radical surgery (total gastrectomy with D2 lymphadenectomy) is indicated, while endoscopic resection may be considered for select cases meeting strict criteria. In locally advanced GSRCC, neoadjuvant platinum‐based chemotherapy combined with surgical resection is recommended, followed by adjuvant chemotherapy with or without hyperthermic intraperitoneal chemotherapy. Immunotherapy may be incorporated for patients exhibiting PD‐L1 combined positive score (CPS) ≥ 5. Metastatic GSRCC is primarily treated with taxane–fluorouracil combination regimens, with immunotherapy added for tumors demonstrating high PD‐L1 expression [8]. The SOX regimen (S‐1 plus oxaliplatin) has emerged as an effective adjuvant therapy for GSRCC [9]. Its antitumor activity stems from dual mechanisms: S‐1 inhibits DNA synthesis through fluorouracil prodrug activation, while oxaliplatin induces DNA crosslinking damage, resulting in synergistic cytotoxicity [10]. Subgroup analysis from the CLASSIC trial [11] demonstrated that GSRCC patients receiving SOX adjuvant chemotherapy after surgery achieved significantly improved 5‐year disease‐free survival (DFS) rates (58% vs. 46%, *p* = 0.03), particularly among patients with lymph node involvement or T3/T4 staging. However, several studies have highlighted the limited efficacy of the SOX regimen in controlling peritoneal micrometastases [12]. Notably, GSRCC patients with > 50% signet‐ring cell component exhibit a 5‐year peritoneal recurrence rate of 52% versus 31% in those with ≤ 50% (*p* < 0.001) [3]. Compared to non‐GSRCC, GSRCC demonstrates markedly reduced chemotherapy sensitivity. Clinical studies report objective response rates (ORRs) of only 22%–30% to fluorouracil or platinum‐based regimens in SRCC patients, significantly lower than the 45%–52% observed in non‐SRCC [13]. Furthermore, postoperative recurrence risk remains substantial, with 50%–65% of patients developing local recurrence or distant metastasis within 5 years despite radical surgery and adjuvant chemotherapy [14].

GSRCC exhibits substantial tumor heterogeneity, with a subset of cancer cells acquiring enhanced migratory and invasive capabilities through epithelial–mesenchymal transition (EMT), thereby promoting tumor recurrence [15]. Accumulating evidence indicates that metformin plays a pivotal role in suppressing EMT in gastric cancer [16]. Experimental studies [17] demonstrate that metformin treatment significantly reduces the expression of the mesenchymal marker vimentin while upregulating the epithelial marker E‐cadherin in a concentration‐dependent manner (all *p* < 0.01), suggesting its potential to impede gastric cancer progression by inhibiting EMT. Furthermore, emerging research reveals [18] that metformin dose‐dependently downregulates both mRNA and protein expression of RAGE and NOX4 in gastric cancer cells. These findings suggest that metformin may reverse EMT by modulating the RAGE/NOXs signaling pathway, consequently attenuating the invasive and metastatic potential of gastric cancer cells. Collectively, these mechanistic insights provide a rationale for exploring metformin as a therapeutic agent in gastric cancer.

Metformin exerts its multitarget antitumor effects through the activation of the AMPK/mTOR pathway, thereby inhibiting tumor cell energy metabolism and proliferation [19, 20]. It also modulates the insulin/IGF‐1 axis to reduce oncogenic signaling, while simultaneously suppressing angiogenesis and improving the tumor microenvironment [21]. In animal studies, metformin administered at doses equivalent to 1‐2 g/day in humans significantly inhibits tumor growth, reducing tumor volume/weight by 40%–60% while decreasing metastatic potential [21–23]. Furthermore, the combination of metformin with FOLFOX regimen (5‐fluorouracil + leucovorin + oxaliplatin) demonstrated significantly enhanced antitumor efficacy, resulting in a 45% reduction in tumor volume compared to monotherapy in nude mice (*p* < 0.05) [24]. In a Phase III RCT, 151 nondiabetic postpolypectomy patients were randomized to metformin (250 mg/day) or placebo for 1 year. Metformin significantly reduced overall adenoma recurrence by 33% (RR = 0.67, 95% CI 0.47–0.97; *p* = 0.034), with a 50% reduction in high‐risk adenomas (≥ 10 mm) [25]. Research indicates that the combination of metformin, the TPF chemotherapy regimen, and apatinib in the treatment of advanced gastric cancer results in improved intestinal microbiota profiles and tumor marker levels, as well as enhanced quality of life scores in the study group compared to the control group (*p* < 0.05) [26].

This article reports a case of GSRCC managed through multidisciplinary team (MDT)–guided individualized therapy. The patient underwent D2 lymphadenectomy followed by adjuvant therapy with the SOXM regimen (oxaliplatin plus S‐1, metformin) and maintenance therapy (apatinib plus S‐1, metformin), achieving 9‐year recurrence‐free survival (RFS).

## 2. Case Presentation

### 2.1. Clinical Presentation

A 60‐year‐old male presented to our hospital on October, in 2016, with a 6‐month history of abdominal pain, bloating, and anorexia. Initial gastroscopy at a referring institution revealed findings suspicious for gastric cancer. Subsequent endoscopic evaluation at our center identified an ulcerative lesion with irregular mucosal elevation along the upper‐middle lesser curvature of the gastric body. Endoscopic ultrasonography (EUS) demonstrated disrupted mucosal layering at the lesion site, accompanied by mild thickening of the muscularis propria. Biopsy confirmed poorly differentiated adenocarcinoma, with immunohistochemistry (IHC) showing HER2 negativity (score 0) (Figure 1). Contrast‐enhanced CT revealed no regional lymphadenopathy or distant metastases. However, relevant preoperative endoscopic and CT image data were missing. Serum carcinoembryonic antigen (CEA) levels were within normal limits (Figure 2). Physical examination noted left upper quadrant tenderness without peritoneal signs or organomegaly. The patient had no significant medical/family history or genetic predisposition to malignancy. The patient was preoperatively diagnosed with primary gastric poorly differentiated adenocarcinoma at TNM Stage IA (pT1N0M0). The patient maintained well‐preserved functional status, with a Karnofsky Performance Status (KPS) score of 90 and an Eastern Cooperative Oncology Group (ECOG) performance status grade of 1.

FIGURE 1Pathological examination results. (a) Hematoxylin‐eosin (H&E) staining before and after the surgery (100 × 10). (b) Immunohistochemical staining after the surgery (40 × 10): Her‐2, 2+; CK8/18, 3+; P53 > 20%+; SYN, −; Ki67, > 80%+; PMS‐2, +; MSH‐6, +; MSH‐2, +; MLH‐1, +.

**Figure:**
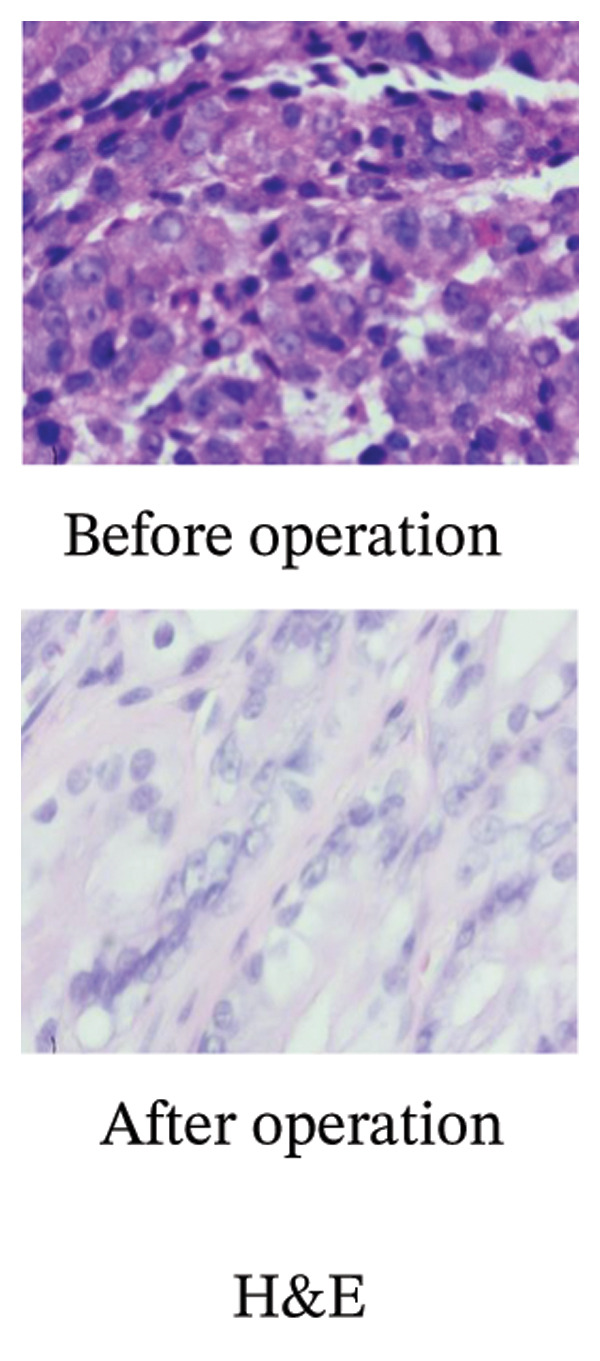
(a)

**Figure:**
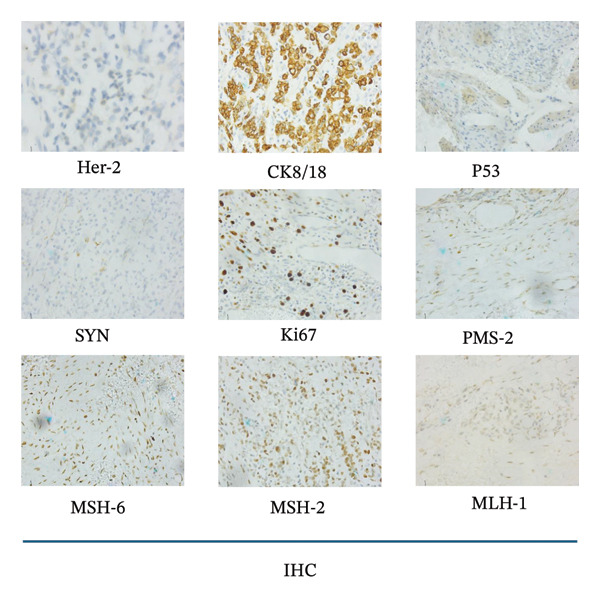
(b)

FIGURE 2Blood examination results. (a) Tumor marker results. (b) Coagulation profile results. (c) Blood routine results. (d) Liver function results and kidney function results.

**Figure:**
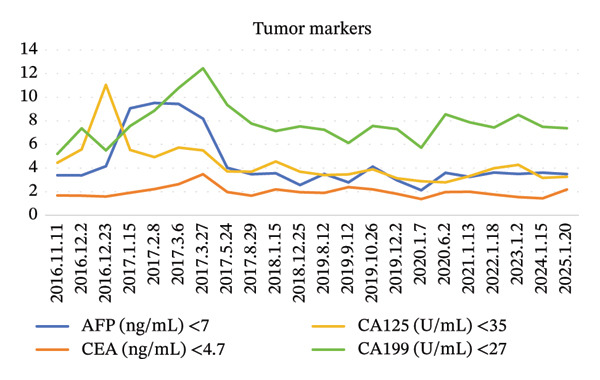
(a)

**Figure:**
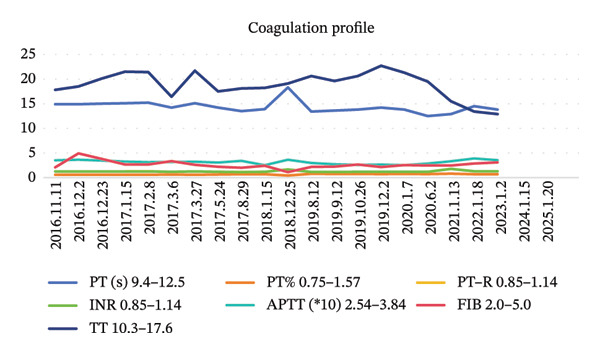
(b)

**Figure:**
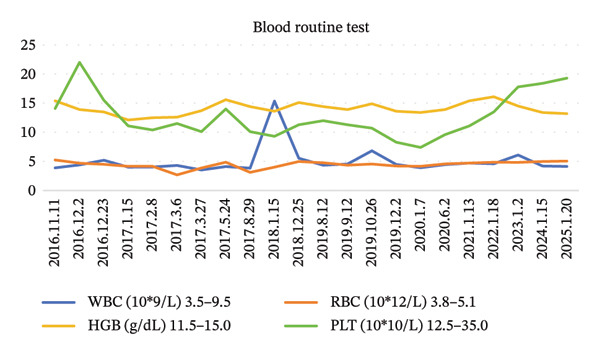
(c)

**Figure:**
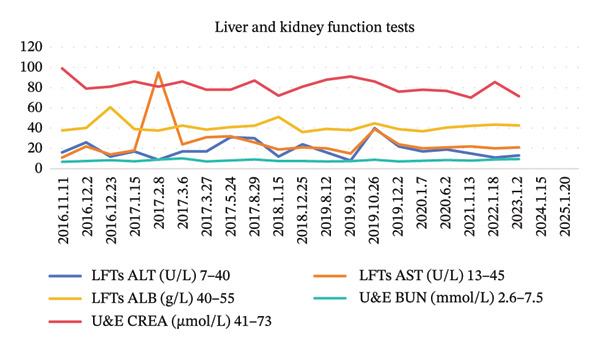
(d)

### 2.2. Treatment Course

A laparoscopic distal radical gastrectomy (D2) with Billroth II anastomosis was performed on November, 2016, under general anesthesia. The surgical specimen revealed SRCC (60%) and partially moderately differentiated adenocarcinoma (40%), with the majority of the cancer confined to the lamina propria of the mucosa, demonstrating local invasion of the muscular layer of the mucosa (pT1b). The vasculature labeled with CD2‐40 and CD31 and nerves remained uninvolved, and no cancer metastasis was observed in the lymph nodes (0/16). Immunohistochemical staining of cancer cells revealed the following markers: CK8/18 (3+), Syn (−), P53 > 20% (+), Her‐2 (2+), Ki67 > 80%+, MLH‐1 (+), MSH‐2 (+), MSH‐6 (+), and PMS‐2 (+) (Figure 1).

At the 30‐day postoperative follow‐up, contrast‐enhanced abdominal CT demonstrated expected post‐distal gastrectomy anatomical changes without anastomotic abnormalities, corroborated by endoscopic findings of a patent and well‐healed anastomosis with normal‐appearing gastric remnant mucosa. Tumor markers remained within normal ranges (CEA: 1.68 ng/mL, CA19‐9: 5.2 U/mL) (Figure 2). Between December 2016 and May 2017, the patient completed six cycles of combination therapy comprising oxaliplatin (85 mg/m^2^, intravenous infusion for 2 h on Day 1, every 21 days), S‐1 (100 mg/m^2^ orally once daily on Days 1–14 of a 21‐day cycle), and metformin (1000 mg daily with dinner). Subsequent surveillance identified vascular endothelial growth factor receptor 2 (VEGFR‐2) (KDR) amplification via genomic testing in June 2019, followed by November 2019 circulating tumor cells (CTC) analysis revealing three isolated chromosomally abnormal CTCs (no microemboli or small CTCs). This prompted therapeutic modification to apatinib mesylate (850 mg daily postprandial) combined with S‐1 (maintained at prior dosage) and continued metformin, and this regimen was maintained for 42 months. Repeat CTC testing in January 2023 showed complete clearance of detectable tumor cells. Standardized supportive care, including gastroprotection, antiemetics, hepatoprotectants, and nutritional support, was administered throughout treatment, contributing to favorable oncologic outcomes.

### 2.3. Adverse Reactions

During chemotherapy, the patient experienced Grade III myelosuppression (NCI‐CTCAE 5.0), characterized by a decrease in white blood cells to 1.8 × 10^9^/L and platelets to 65 × 10^9^/L. To address this, the patient was administered recombinant human granulocyte colony‐stimulating factor (rhG‐CSF) concurrently with interleukin‐11 (IL‐11) for thrombocytopenia management, along with a stratified nursing intervention. Complete recovery of the peripheral blood profile was observed 21 days after the completion of the chemotherapy cycle. Mild gastrointestinal reactions may occur in the initial stage of oral metformin administration, and vitamin B12 should be supplemented preventively during long‐term medication.

### 2.4. Follow‐Up

**FIGURE 3:**
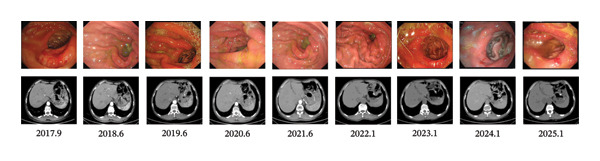
Follow‐up contrast‐enhanced CT and endoscopic images.

**FIGURE 4:**
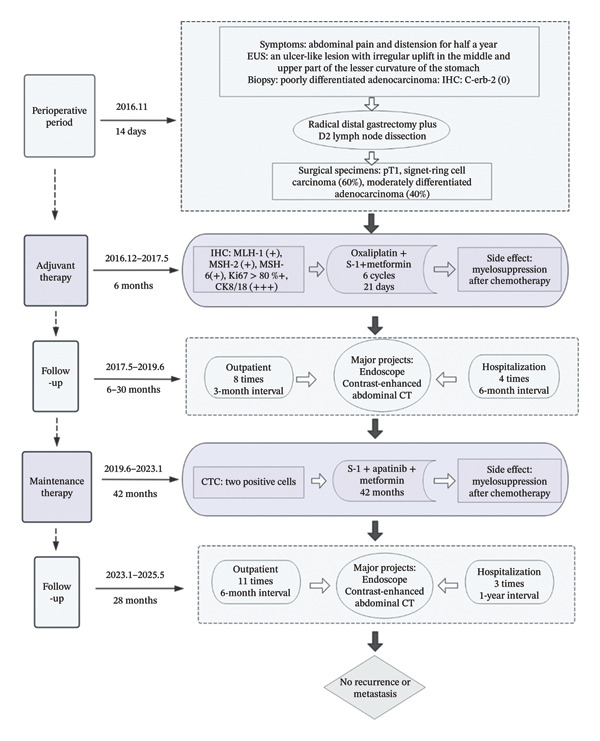
Treatment timeline and drug regimen.

The patient was placed on a structured surveillance protocol with quarterly follow‐up evaluations during the first postoperative year, transitioning to semiannual assessments from years 2 to 5, followed by annual monitoring through 6–9 years (Figure 3). On August 29, 2017, a follow‐up endoscopy was performed, and a biopsy was taken, which revealed chronic inflammation of the anastomotic mucosa. Tumor markers showed CA199, CEA, and CA125 within normal ranges, while AFP was 9.5 ng/mL (reference range: < 7 ng/mL). This slight elevation of AFP may be attributed to mild impairment of liver metabolism caused by chemotherapy. On June 12, 2018, endoscopy showed a post‐subtotal gastrectomy (Billroth II) state, with intact residual gastric mucosa, a smooth anastomosis, and normal mucosal color. On June 12, 2019, contrast‐enhanced abdominal CT indicated a slight enlargement of the lesion in the space between the pancreas and splenic hilum. Endoscopy revealed congestion, redness, and partial elevation of the anastomotic mucosa. Symptomatic treatment was administered, and further observation was continued. On June 2, 2020, CT showed a reduction in the lesion in the space between the pancreas and splenic hilum compared to previous scans. On January 13, 2021, CT scan indicated mild thickening of the anastomosis, with the lesion in the space between the pancreas and splenic hilum remaining stable compared to prior images. Endoscopy showed an unobstructed gastrojejunal anastomosis with localized redness of the mucosa. On January 20, 2022, CT scan showed no obvious abnormalities at the anastomosis. On May 30, 2022, endoscopy diagnosed bile reflux gastritis. From January 2023 to January 2025, follow‐up CT scans and endoscopies showed no significant abnormalities. The quality of life questionnaire (QLQ‐C30) yielded a score of 92, indicating a high level of functionality and well‐being. The RFS of the patients was 9 years and reached the clinical cure standard (Figure 4).

## 3. Discussion

**FIGURE 5:**
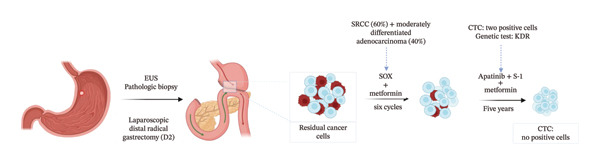
Mechanism diagram of the interactions between drugs (created in BioRender [2025]—https://BioRender.com).

GSRCC is characterized by high malignancy, early metastasis (particularly peritoneal metastasis), and resistance to conventional therapies, with its 5‐year survival rate significantly lower than that of other gastric cancer subtypes. The key strength of this case lies in the implementation of a comprehensive treatment strategy encompassing “radical surgery–adjuvant chemotherapy–long‐term maintenance therapy” (Figure 3). Laparoscopic distal gastrectomy combined with D2 lymph node dissection enables complete resection of the primary tumor and thorough clearance of regional lymph nodes within the framework of minimally invasive surgery, thereby establishing a solid foundation for subsequent systemic treatment. Six months of adjuvant therapy with oxaliplatin, S‐1, and metformin effectively reduces tumor burden and addresses the chemosensitivity of both histological components in the mixed pathology (60% GSRCC and 40% moderately differentiated adenocarcinoma). The subsequent 42‐month oral administration of apatinib, S‐1, and metformin establishes a multimechanistic synergistic regimen involving “chemotherapy–targeted therapy–metabolic regulation,” continuously suppressing the activity of residual tumor cells. This sequential treatment strategy may reduce the high recurrence risk linked to GSRCC through long‐term maintenance therapy, thus providing a potential whole‐course management model for highly aggressive malignancies (Figure 5).

In this particular case, the patient received adjuvant therapy at 2 years after surgery, and follow‐up CT scans indicated a slight enlargement of the lesion in the pancreaticosplenic hilum space. Endoscopic examination revealed congestion, erythema, and partial elevation of the anastomotic mucosa. Genomic testing was subsequently conducted, which identified VEGFR‐2 (KDR) amplification. Furthermore, CTC analysis detected three isolated CTCs with chromosomal abnormalities. The patient’s pathological type was confirmed as SRCC (60%) mixed with partially moderately differentiated adenocarcinoma (40%). A comprehensive analysis indicated the presence of occult micrometastases or chemoresistant tumor subclones, suggesting a high risk of tumor recurrence [27]. Following a MDT discussion, an individualized therapeutic regimen was adopted: apatinib mesylate combined with S‐1 and continuous metformin administration. These findings suggest that an intensified step‐up maintenance strategy combining S‐1 with apatinib, an antiangiogenic agent, may serve as a potential therapeutic option for long‐term disease control.

Based on this case and existing evidence, the 42‐month combination of S‐1 and apatinib and metformin enteric‐coated tablets represents an innovative long‐term maintenance strategy for GSRCC post‐surgery, overcoming the temporal constraints of conventional adjuvant therapy (typically less than 1 year) (Figure 5). Studies have demonstrated that the gemcitabine component in S‐1 can continuously inhibit dihydropyrimidine dehydrogenase and significantly prolong the area under the plasma concentration‐time curve of 5‐fluorouracil [28]. We therefore hypothesize that long‐term administration of S‐1 may effectively target and eliminate the signet‐ring cell subpopulations characterized by the high expression of thymidylate synthase. Meanwhile, apatinib exerts a potent tyrosine kinase inhibitory effect to abrogate VEGFR‐2–mediated angiogenesis and thereby suppresses the proliferation of tumor cells [29]. Clinically, Phase II clinical trials have demonstrated that the apatinib plus S‐1 doublet regimen, as second‐line therapy for advanced gastric cancer, achieved an ORR of 17.2% and a disease control rate of 75.8%, which validates its favorable efficacy and tolerability in this setting [30]. Building upon this foundation, we incorporated metformin extended‐release tablets into the treatment regimen. Metformin exerts multitarget synergistic effects by regulating energy metabolism, key signaling pathways, and the tumor microenvironment [19]. Research has confirmed that metformin exhibits significant inhibitory effects in both gastric cancer stem cells and patient‐derived tumor xenograft models, achieving a 54% reduction in tumor volume while suppressing cancer stem cells’ self‐renewal capacity by over 70% [31]. Metformin, as a classic hypoglycemic drug, has been confirmed in recent years to have antitumor potential independent of its hypoglycemic effect [20], but its application in long‐term maintenance treatment of GSRCC has not yet received widespread attention.

Despite the success of this case, several limitations warrant consideration. The single‐arm study design precludes definitive causal inferences, and the proposed 10‐year maintenance therapy duration requires validation through randomized controlled trials.

## 4. Conclusion

This case received a comprehensive, stepwise treatment regimen tailored to the highly invasive nature of GSRCC. Dynamic CTC monitoring allowed precise therapeutic adjustments, and combined use of metformin during maintenance may contribute to long‐term disease control, with the patient achieving a 9‐year RFS. As this is a single case report, the treatment strategy has only potential reference value and may generate hypotheses for future studies.

## Author Contributions

Lingzhi Liao and Hao Chen: conceptualization and writing–review and editing. Yifan Li and Dan Zou: writing–original draft. Jing Liu and Guogui Wang: visualization and revision. Jingwei Ma, Yali Li, and Yile Qi: investigation. Bin Ma and Jiahui Ma: supervision.

## Funding

This research was supported by the National Natural Science Foundation of China (82160129).

## Ethics Statement

Ethical approval to report this case was obtained from the Medical Ethics Committee of the Second Hospital & Clinical Medical School, Lanzhou University (2024A‐660). Written informed consent was obtained from each patient.

## Consent

Written informed consent was obtained from the patient for publication of clinical details and clinical images.

## Conflicts of Interest

The authors declare no conflicts of interest.

## Data Availability

All relevant clinical details are included within this manuscript. Additional information can be obtained from the corresponding author upon reasonable request, while maintaining patient confidentiality.
